# Effect of Orally Administered* Atractylodes macrocephala* Koidz Water Extract on Macrophage and T Cell Inflammatory Response in Mice

**DOI:** 10.1155/2018/4041873

**Published:** 2018-08-07

**Authors:** Tae-Kyung Kwak, Hyung-Seok Jang, Mi-Gi Lee, Young-Sung Jung, Dae-Ok Kim, Yoon-Bum Kim, Jong-In Kim, Hee Kang

**Affiliations:** ^1^Graduate School of East-West Medical Science, Kyung Hee University, Yongin 17104, Republic of Korea; ^2^Jang Hyung-Seok Korean Medicine Clinic, Seoul 06524, Republic of Korea; ^3^Bio-Center, Gyeonggido Business and Science Accelerator, Suwon 16229, Republic of Korea; ^4^Department of Food Science and Biotechnology, Kyung Hee University, Yongin 17104, Republic of Korea; ^5^Department of Oriental Dermatology, College of Korean Medicine, Kyung Hee University, Seoul 02447, Republic of Korea; ^6^Department of Acupuncture and Moxibustion Medicine, Kyung Hee University, Seoul 02447, Republic of Korea

## Abstract

The rhizome of* Atractylodes macrocephala* Koidz (AM) is a constituent of various Qi booster compound prescriptions. We evaluated inflammatory responses in macrophages and T cells isolated from mice following oral administration of AM water extract (AME). Peritoneal exudate cells were isolated from thioglycollate-injected mice and alterations in scavenger receptors were examined. Peritoneal macrophages were stimulated with lipopolysaccharide (LPS). Serum cytokine responses to intraperitoneal LPS injection were also evaluated. Splenocytes were isolated and their composition and functional responses were measured. The content of atractylenolide I and atractylenolide III, known anti-inflammatory ingredients, in AME was 0.0338 mg/g extract and 0.565 mg/g extract, respectively. AME increased the number of SRA(+)CD11b(+) cells in response to thioglycollate. Peritoneal macrophages isolated from the AME group showed no changes in inflammatory markers such as tumor necrosis factor- (TNF-) *α*, interleukin- (IL-) 6, inducible nitric oxide synthase, and cyclooxygenase-2 but exhibited a decrease in CD86 expression. Interestingly, AME decreased the serum levels of TNF-*α* and IL-6 upon intraperitoneal injection of LPS. Regarding the adaptive immune system, AME increased the CD4(+) T cell population and major histocompatibility complex class II molecule expression in the spleen, and cultured splenocytes from the AME group showed increased production of IL-4 concurrent with decreased interferon-*γ* production during T cell activation. AME promoted the replenishment of peritoneal macrophages during the inflammatory response but its anti-inflammatory activity did not appear to be mediated by the modulation of macrophage activity. AME also altered the immune status of CD4 T cells, promoting the Th2 response.

## 1. Introduction

Inflammation is a protective response to eliminate harmful stimuli, and immune cells are the major participants in this process. Depending on the modality of antigen recognition and the capacity to generate memory response, immune cells are divided into the innate immune system and the adaptive immune system [1]. Innate immune cells such as macrophages and dendritic cells react instantly to antigen with limited receptor specificity [1]. Adaptive immune cells, consisting of T cells and B cells, are antigen-specific, initiate a response to antigen that has entered the peripheral lymphoid tissue, and generate a memory response [1]. The innate immune cells are principal players in the early stages of inflammation, but over time, adaptive immune cells take over.

Tissue resident macrophages play a key role in immunity and tissue integrity [2]. Most tissue macrophages are derived from embryonic precursors [3]. Under steady-state conditions their populations are maintained through their longevity and by local proliferation, and some macrophages are replenished by blood monocyte-derived cells [3]. During inflammation, bone marrow-derived monocytes are recruited to the site and differentiate into macrophages [3]. Macrophages eliminate pathogens and antigens through phagocytosis and induce inflammatory responses by producing cytokines and enzymes such as tumor necrosis factor- (TNF-) *α*, interleukin- (IL-) 6, inducible nitric oxide synthase (iNOS), and cyclooxygenase- (COX-) 2. In addition, macrophages are one type of professional antigen presenting cells (APCs) that present antigens to T cells [4, 5].

T cells, which mainly consist of CD4 T cells and CD8 T cells, are activated when T cell receptors (TCRs) contact antigenic peptides bound by major histocompatibility complex (MHC) molecules on APCs [6]. CD4 T cells, which account for more than two-thirds of T cells, can be differentiated into various effector T helper (Th) cells such as Th1, Th2, Th17, T follicular helper, and T regulatory cells [7]. Among these subsets, Th1 and Th2 cells were the first types to be defined. Th1 cells secrete high levels of interferon- (IFN-) *γ* and are efficient in the defense against intracellular pathogens by activating macrophages whereas Th2 cells secrete interleukin- (IL-) 4, IL-5, and IL-13 and protect the host from helminth infection by recruiting eosinophils and mast cells [7]. Although these T helper cells are important for host defense, chronic activation of any Th cell type can cause immune-mediated disorders. Th1 cells play a critical role in organ-specific autoimmunity and chronic inflammatory disorders and Th2 cells are responsible for allergic inflammation [7].

The rhizome of* Atractylodes macrocephala* Koidz (AM), belonging to the Compositae, has been used for the treatment of functional defects in the digestive system such as loss of appetite, abdominal distention, and diarrhea. According to traditional Chinese medicine, AM invigorates Qi by resolving abnormal retention of fluid in the gastrointestinal tract. AM is a constituent of various Qi booster compound prescriptions. In traditional Chinese medicine, one of the essential functions of Qi is defense. For this reason, Qi boosting herbs are thought to enhance the immune system. Since Qi boosting herbs are taken on a preventive basis to improve the immune status of individuals without overt defects, it is necessary to evaluate how the immune system may be altered in normal individuals following the administration of AM. Despite its frequent use, there have been few studies to explore the effects of AM on the immune system.

AM contains several bioactive sesquiterpenoids such as atractylenolide I, atractylenolide II, and atractylenolide III and polyacetylenes [8].* In vitro* treatment of macrophages with atractylenolide I, atractylenolide III, and some polyacetylenic compounds inhibited lipopolysaccharide- (LPS-) induced TNF-*α* and iNOS expression [9, 10]. Oral administration of these lipid-soluble components showed anti-inflammatory activity in mice [11, 12]. However, the majority of traditional herbal preparations are water-based decoctions, which results in a low yield of pharmacologically active lipid-soluble components. Furthermore, polyacetylenes can be easily destroyed in boiling water. Therefore, we wanted to address whether anti-inflammatory responses occur in macrophages isolated from mice given AM extracted in boiling water (AME). We also examined the effect of AME on the serum inflammatory response. Finally, we examined the composition and functional response of splenocytes for any alteration in the adaptive immune system after AME supplementation.

## 2. Materials and Methods

### 2.1. Preparation of Sample

AM originating from Eusung (South Korea) was purchased from E-Pulip Co., Ltd. (Lot. EPL1356-4) (Seoul, South Korea). A voucher specimen (# 2013-AM) was deposited in the Laboratory of Herbal Immunology, Kyung Hee University. Briefly, 100 g of sample was ground, extracted with 1 L of deionized water (DW) in a reflux apparatus and heating mantle for 2 h at 95°C, and filtered through Whatman number 2 filter paper (Whatman International, Kent, England). The extract was concentrated using a rotary evaporator and freeze-dried under vacuum. The yield of AME was 37.7%. For high-performance liquid chromatography (HPLC) analysis, 0.4 g of AME was dissolved in 10 ml of DW and sonicated for 5 min at 25°C. The extract was added to ethyl acetate, shaken to mix, and allowed to stand for 1 min. The upper layer of ethyl acetate was transferred and this procedure was repeated three times. The final ethyl acetate layer was concentrated and freeze-dried.

### 2.2. HPLC

Samples were analyzed by a reverse-phase HPLC system (Shimadzu 20A, Kyoto, Japan) that consisted of an autosampler (SIL-20A), a binary pump (LC-20AD), and a photodiode array detector (SPD-20A) and was equipped with a YMC-Triart C18 column (5 *μ*m × 4.6 mm × 250 mm) (YMC, Kyoto, Japan). Gradient flows for the two-solvent system (solvent A, 0.05% phosphoric acid in water; solvent B, acetonitrile) were as follows: 85% A/15% B at 0 min, 85% A/15% B at 5 min, 50% A/50% B at 15 min, 50% A/50% B at 20 min, 40% A/60% B at 25 min, 40% A/60% B at 30 min, 15% A/85% at 35 min, 15% A/85% at 40 min, 85% A/15% at 42 min, and 85% A/15% at 45 min. The flow rate of the mobile phase was 1.0 ml/min with an injection volume of 10 *μ*l. Detection was performed at 220 nm for atractylenolide III (Sigma, St. Louis, MO, USA) or at 280 nm for atractylenolide I (Sigma).

### 2.3. Animals

Seven-week-old male Balb/c mice were obtained from SamTaco (Osan, South Korea) and housed in a temperature- and humidity-controlled pathogen-free animal facility with a 12-h light-dark cycle. All animals underwent 1 week of adjustment prior to experiments. Doses were determined using a calculation extrapolated from the difference in body surface area between a mouse and a human [13]. The recommended dose of AM for a 60 kg adult human is 8-24 g of raw plant per day or 3-9 g of extract per day (based on the extraction yield in this study). The dose for mouse can be determined as follows: a human equivalent dose of 50-150 mg/kg × 12.3 (the conversion coefficient) = a mouse dose of 615-1,845 mg/kg. Based on this dose range, we chose doses of 500 mg/kg and 2,500 mg/kg for this study. Animals were randomly allocated to experimental groups. AME was given via oral gavage once daily for 10 days. There were no differences in body weight among groups during the experimental period. The animal protocol was approved by the Institutional Animal Care and Use Committee at Kyung Hee University (KHUASP(SE)-15-012), and mice were cared for according to US National Research Council for the Care and Use of Laboratory Animals (1996) specifications.

### 2.4. Macrophage Preparation

For macrophage isolation, mice were injected intraperitoneally with 2 ml of 3.5% sterile thioglycollate (BD, Sparks, MD, USA) 4 days before sacrifice. At the end of the experiment, mice were sacrificed by cervical dislocation and peritoneal exudate cells were aseptically isolated by peritoneal lavage with cold DMEM (Hyclone, Logan, UT, USA) containing 10% fetal bovine serum (FBS; Hyclone) and 1% penicillin-streptomycin. After centrifugation, cells were resuspended and counted using a TC20 Cell Counter (Bio-Rad Laboratories, Hercules, CA, USA).

### 2.5. Splenocyte Preparation

For splenocyte isolation, spleens were aseptically obtained at the end of the experiment. After disrupting the spleen between glass slides in RPMI 1640 (Hyclone) with 1% FBS and 1% penicillin-streptomycin, the cells were filtered through a 70-*μ*m cell strainer. After centrifugation, red blood cells were lysed using BD PharmLyse lysing buffer (BD Biosciences, San Diego, CA, USA). Cells were resuspended in RPMI 1640 with 10% FBS and 1% penicillin-streptomycin and counted using a T20 Cell Counter.

### 2.6. Intraperitoneal Injection of LPS

Mice were intraperitoneally injected with 1.3 mg/kg LPS (serotype 055:B5, Sigma) at the end of the experiment. After 1 h, mice were anesthetized with ether and blood was collected by cardiac puncture. Serum was obtained and stored at −20°C until analysis.

### 2.7. Cell Culture

Peritoneal exudate cells were plated in 6-well plates or 60-mm dishes and incubated overnight at 37°C. After removal of nonadherent cells, attached cells were stimulated with 100 ng/ml LPS for 24 h. Supernatant and cells were collected for subsequent assays. Splenocytes were plated in 24-well plates and stimulated with 2 *μ*g/ml anti-CD3 antibody (BD Biosciences) for 48 h. Supernatant was collected for cytokine analysis.

### 2.8. Flow Cytometry

Cells were washed twice in phosphate buffered saline (PBS) and resuspended at 1 × 10^6^ cells/ml in FACS buffer (PBS/0.1% NaN_3_/1% FBS). Cells were blocked with rat anti-mouse CD16/CD32 antibody (BD Biosciences) at 4°C for 5 min and then stained with fluorescein-conjugated anti-mouse SR-AI, PE-conjugated anti-mouse LOX1 (R&D Systems, Minneapolis, MN, USA), PE-conjugated anti-mouse CD36, FITC-conjugated CD11b, PE-conjugated anti-CD11b, PE-conjugated anti-mouse CD86, FITC-conjugated anti-mouse CD4, PE-conjugated anti-mouse CD8a, FITC-conjugated anti-mouse CD19, and FITC-conjugated anti-mouse IA/IE (BD Biosciences) (all antibodies were diluted 1:100) for 30 min on ice in the dark. Matched isotype antibodies were used to show nonspecific binding. The cells were washed and resuspended in FACS buffer. A total of 10,000 events were acquired on a Navios flow cytometer (Beckman Coulter, La Brea, CA, USA), and the data were processed using Kaluza software (Beckman Coulter).

### 2.9. Cytokine Analysis

The levels of TNF-*α*, IL-6, IFN-*γ*, and IL-4 in supernatants and sera were determined using BD OptEIA mouse ELISA sets (BD Biosciences) according to the manufacturer's protocol.

### 2.10. Proliferation Assay

Splenocytes (4 × 10^5^) in 96-well plates were stimulated with soluble anti-CD3 mAb (2 *μ*g/ml) for 48 h. Cell proliferation was determined using the CellTiter96 One Solution Cell Proliferation assay kit (Promega, Madison, WI, USA).

### 2.11. RNA Isolation and Real-Time PCR

Total RNA was isolated using a FavorPrep Total RNA Purification Kit (Favorgen Biotech, Pingtung, Taiwan), and cDNA was reverse-transcribed using a High Capacity RNA-to-cDNA kit (Applied Biosystems, Foster City, CA, USA). Diluted cDNA was mixed with Power SYBR Green PCR Master mix (Applied Biosystems) and 2 pmol of primers specific for iNOS, COX2, or GAPDH. Amplification of cDNA was performed using a StepOnePlus real-time PCR system (Applied Biosystems). After initial heat denaturation at 95°C for 10 min, PCR conditions were set at 95°C for 15 sec and 60°C for 1 min for 40 cycles. For each PCR, a corresponding mRNA sample without reverse transcription was included as a negative control. Quantification of cDNA copy number was achieved using a standard curve.

### 2.12. Statistical Analysis

Data were presented as mean standard error of the mean (SEM). Two-sided Student's t-test or two-way analysis of variance was applied to compare differences between groups. If the statistical analysis showed that differences between multiple groups were significant, Tukey* post hoc* test was used for further comparison. All statistical analyses were performed with IBM SPSS 22.0 version software (IBM, Chicago, IL, USA).* P*-values less than 0.05 were considered significant.

## 3. Results

### 3.1. Content of Atractylenolide I and Atractylenolide III in AME

**Figure 1:**
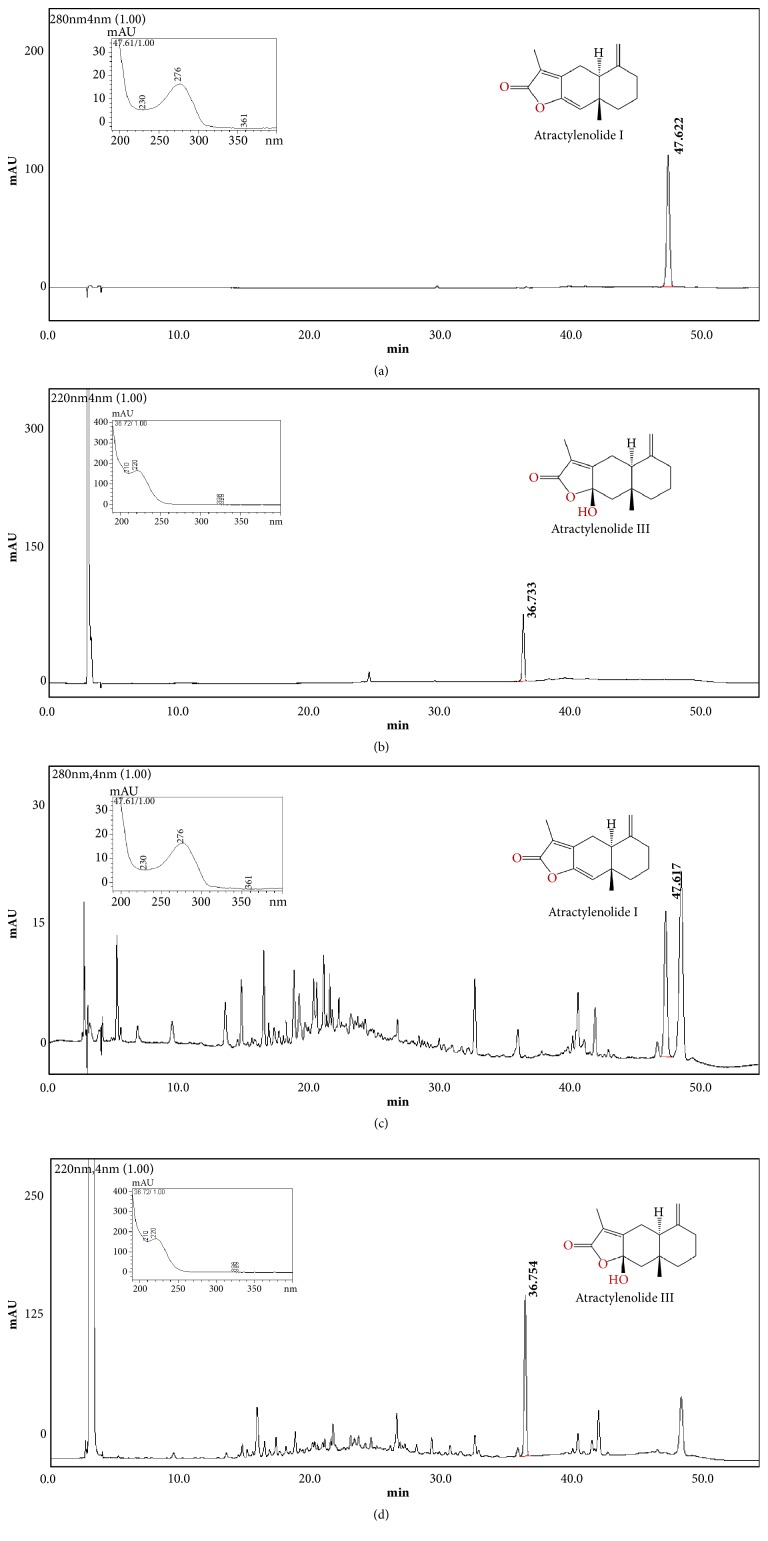
HPLC chromatograms of* Atractylodes macrocephala* water extract (AME). (a, b) Standard markers. (c, d) AME.

Among the known quality control markers, atractylenolide I and atractylenolide III are verified anti-inflammatory compounds* in vitro* [9]. The ethyl acetate fraction from AME was tentatively identified using a spiked input of authentic standards with comparison of retention times and UV-visible spectral patterns. The HPLC chromatograms are shown in Figure 1. The content of atractylenolide I and atractylenolide III in AME was 0.0338 mg/g extract and 0.565 mg/g extract, respectively.

### 3.2. Effect of Oral Administration of AME on Scavenger Receptor Expression in Mouse Peritoneal Exudate Cells

**Figure 2:**
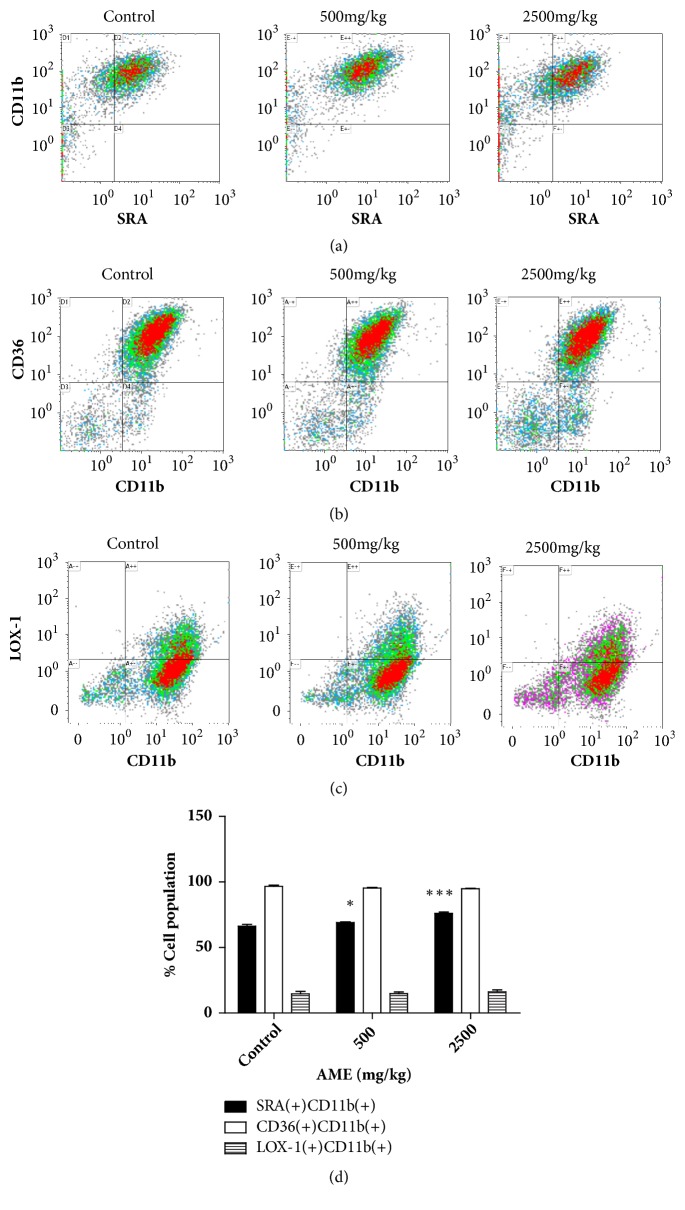
Scavenger receptors expressed by peritoneal exudate cells after oral administration of AME. Mice were orally given AME (500 or 2500 mg/kg) for 10 days. Peritoneal exudate cells were isolated from thioglycollate-injected mice and double-stained with FITC-conjugated anti-SRA and PE-conjugated anti-CD11b Abs (a), FITC-conjugated anti-CD11b and PE-conjugated anti-CD36 Abs (b), or FITC-conjugated anti-CD11b and PE-conjugated anti-LOX-1 Abs (c). Cells were analyzed using flow cytometry and representative dot plots are shown. (d) Bars represent mean ± SEM (n=6). *∗ P* <0.05, *∗∗∗ P*<0.005 versus control.

Intraperitoneal injection of thioglycollate is commonly used to induce sterile peritonitis and enrich peritoneal macrophages from mice in laboratories [14]. The majority of peritoneal macrophages are derived from blood monocytes [15]. We collected peritoneal exudate cells from AME-treated mice using this method. CD11b was used as a marker for macrophages. Scavenger receptors such as SRA, CD36, and LOX-1 are upregulated during monocyte-to-macrophage differentiation [16–18]; therefore we examined the expression of these proteins. SRA, CD36, and LOX-1 were almost exclusively expressed in CD11b(+) cells (Figures 2(a)–2(c)). The percentage of SRA(+)CD11b(+) cells in the control group was 66%, and treatment with 500 mg/kg and 2,500 mg/kg AME significantly increased this population to 69% and 76%, respectively. The frequencies of CD36(+)CD11b(+) and LOX-1(+)CD11b(+) cell populations in the control group were 95% and 14%, respectively, and AME induced no significant changes in both populations. The increase in the SRA(+)CD11b(+) cell population indicates that AME can stimulate the differentiation of blood monocytes into macrophages in response to thioglycollate.

### 3.3. Effect of Oral Administration of AME on Surface CD86 Expression in LPS-Stimulated Macrophages

**Figure 3:**
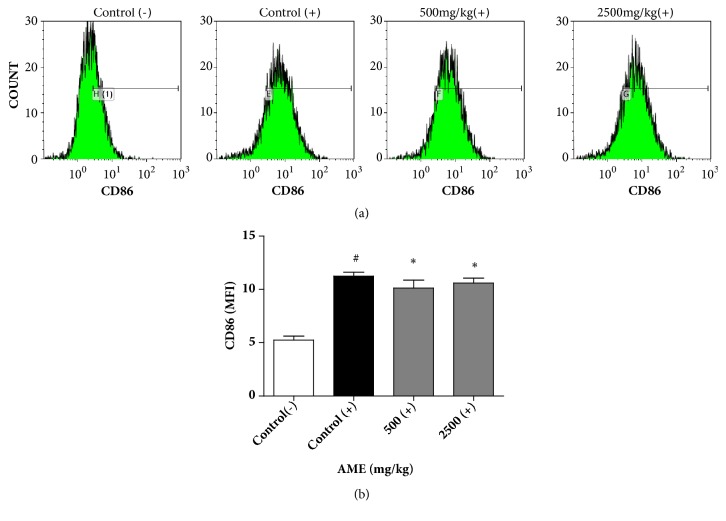
Effect of AME on the surface expression of costimulatory molecules in LPS-stimulated macrophages. Peritoneal macrophages isolated from the control and AME groups were stimulated with 100 ng/ml LPS for 24 h and then stained with PE-conjugated anti-CD86 antibody. (a) Representative histograms are shown. (b) Bars represent mean ± SEM (n=6). (-): without LPS, (+): LPS treatment. MFI: mean fluorescence intensity. #* P*<0.005 versus control (-), *∗ P*<0.05 versus control (+).

Costimulatory molecules such as CD86 on macrophages are required to strengthen the crosstalk between macrophages and Th cells [6]. Peritoneal macrophages isolated from AME-treated mice were stimulated with LPS for 24 h and the membrane expression of CD86 was measured using flow cytometry. Stimulation with LPS increased the mean fluorescence intensity of CD86 from 5.24 to 11.24 (Figure 3). The mean fluorescence intensity of CD86 in the 500 and 2,500 mg/kg groups significantly decreased to 10.14 and 10.59, respectively. These results indicate that AME may affect the interaction between macrophages and Th cells.

### 3.4. Effects of Oral Administration of AME on the Inflammatory Cytokine Response in Macrophages and Serum

**Figure 4:**
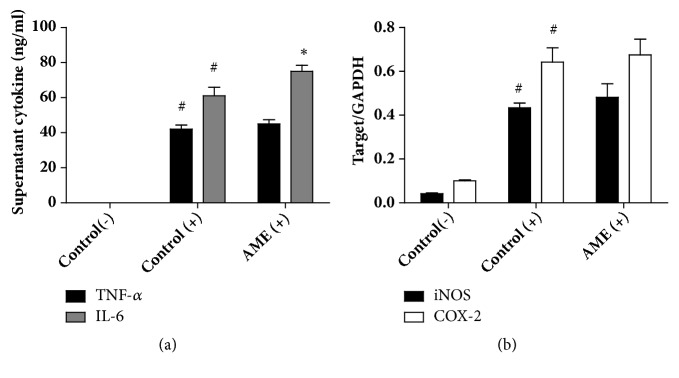
Effect of AME on inflammatory cytokines and enzymes in LPS-stimulated macrophages. Macrophages isolated from control or AME group (2500 mg/kg) were stimulated with LPS (100 ng/ml) for 24 h. (a) The levels of tumor necrosis factor- (TNF-) *α* and interleukin- (IL-) 6 in supernatant were determined by ELISA. (b) Quantitative PCR was used to measure the expression of iNOS and COX-2 genes. Target gene expression was normalized to GAPDH expression. Data represent mean ± SEM (n=6). (-): without LPS, (+): LPS treatment. #* P*<0.005 versus control (-), *∗ P*<0.05 versus control (+).

**Figure 5:**
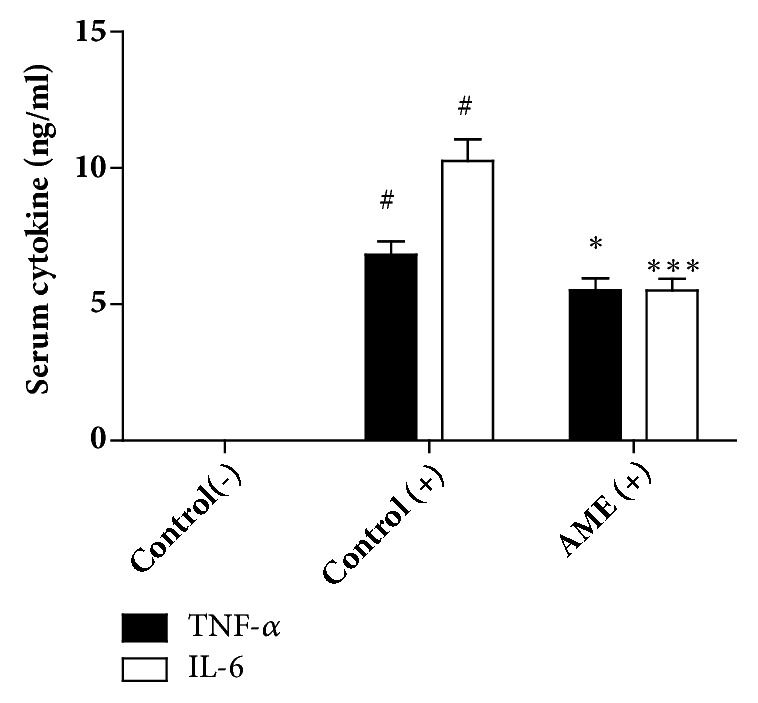
Effects of oral administration of AME on serum inflammatory responses following intraperitoneal injection of LPS. AME (2500 mg/kg) was orally administered to mice for 10 days. Serum was obtained 1 h after intraperitoneal injection of LPS (1.3 mg/kg) and the serum levels of TNF-*α* and IL-6 were determined by ELISA. Data represent mean ± SEM (n=10). (-): without LPS, (+): LPS treatment. #* P*<0.001 versus control (-), *∗ P* <0.05, *∗∗∗ P* <0.001 versus control (+).

We first examined whether oral administration of AME affects the inflammatory response of macrophages. Peritoneal macrophages from the control or high-dose AME group were stimulated with LPS for 24 h and production of TNF-*α* and IL-6 in the supernatant was measured. There was no difference in the level of TNF-*α* secretion between control and AME groups but the level of IL-6 was increased in the AME group (Figure 4(a)). We also found that AME did not induce any alterations in iNOS and COX-2 gene expression in cells stimulated with LPS (Figure 4(b)). Next, we examined the systemic response of AME-treated mice to intraperitoneal LPS stimulation. AME decreased serum levels of TNF-*α* and IL-6 by 20% and 47%, respectively (Figure 5). These findings indicate that the anti-inflammatory activity of AME may occur independently of the modulation of macrophages.

### 3.5. Effects of Oral Administration of AME on Splenic T Cell and B Cell Populations and MHC II Expression

**Figure 6:**
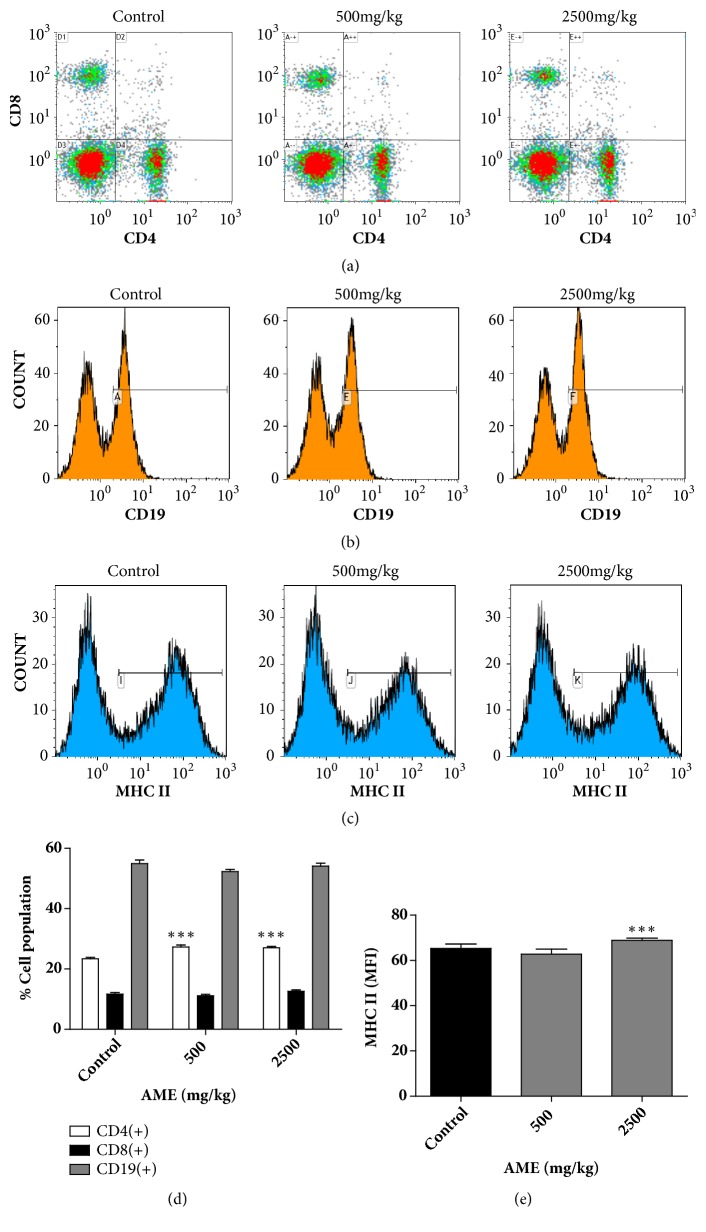
Effects of oral administration of AME on composition of the adaptive immune system in the spleen. Splenocytes were isolated from control or AME groups and double-stained with FITC-conjugated anti-CD4 antibody and PE-conjugated anti-CD8 antibody (a), FITC-conjugated anti-CD19 antibody (b), or FITC-conjugated anti-MHC II antibody (c) and evaluated using flow cytometry. (a-c) Representative dot blots or histograms are shown. (d-e) Bars represent mean±SEM (n=6). *∗∗∗ P* <0.001 versus control.

To determine whether oral administration of AME alters adaptive immune cells, we analyzed the percentages of splenic CD4 and CD8 T cells and B cells in the control and AME groups. The CD4(+) T cell population significantly increased from 23.4% to 27.2% and 26.9% in the 500 and 2,500 mg/kg groups, respectively (Figures 6(a) and 6(d)). No differences were observed in CD8 T cell and B cell populations (Figures 6(a), 6(b), and 6(d)). MHC class II molecules are required for the presentation of antigens to CD4 T cells. We analyzed the splenic expression of the mouse MHC class II molecules IA/IE and found that the mean fluorescence intensity of MHC II molecules significantly increased from 65.3 to 68.9 in the 2,500 mg/kg AME group (Figures 6(c) and 6(e)). These findings suggest that AME induces alterations in the adaptive immune system.

### 3.6. Effects of Oral Administration of AME on T Cell Proliferation and Th1/Th2 Cytokine Response in Splenocytes

**Figure 7:**
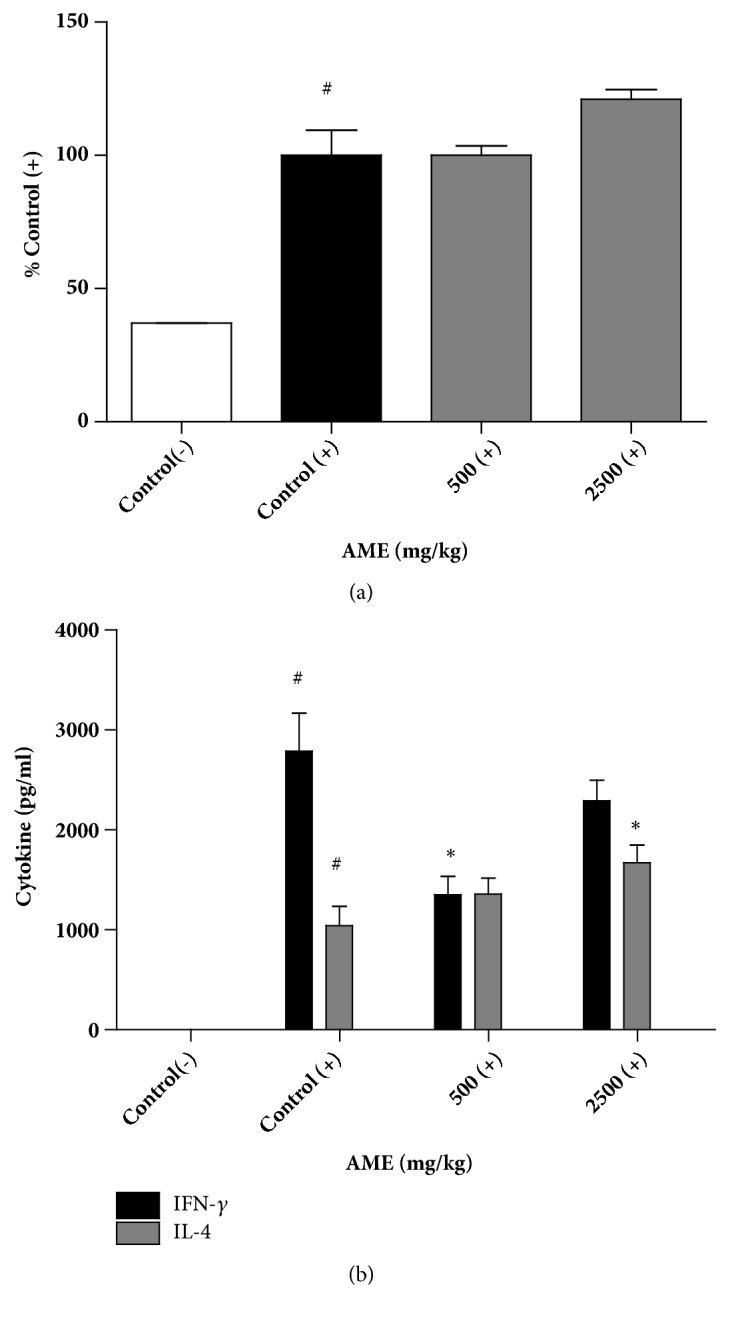
Effects of AME on the proliferation and cytokine secretion of activated splenic T cells. Splenocytes isolated from control and AME groups were cultured and stimulated with anti-CD3 antibody (2 *μ*g/ml) for 48 h. (a) The proliferative response of splenocytes was determined using the MTS assay. (b) Cytokine secretion at 48 h of stimulation was measured by ELISA. Bars represent the mean±SEM (n=6). (-): without anti-CD3 antibody, (+): anti-CD3 antibody treatment. #* P* <0.001 versus control (-), *∗ P* <0.05 versus control (+).

We investigated the function of splenic T cells following AME treatment. Splenocytes isolated from control or AME groups were stimulated with anti-CD3 antibody, a mitogen that activates the whole population of T cells irrespective of antigen receptor specificity. Treatment with anti-CD3 antibody for 48 h increased optical density 2.6-fold as measured by MTS assay. There was no difference in proliferation induced by anti-CD3 antibody between control and AME groups (Figure 7(a)). IFN-*γ* and IL-4 are representative cytokines for Th1 and Th2 cells, respectively. We evaluated the secretion of IFN-*γ* and IL-4 in splenocytes stimulated with anti-CD3 antibody. A significant reduction in IFN-*γ* secretion was observed in the 500 mg/kg AME group, whereas IL-4 secretion was significantly increased in the 2,500 mg/kg group (Figure 7(b)). Although no dose-dependent effect was observed, AME tended to promote the Th2 response.

## 4. Discussion

In traditional Chinese medicine, Qi boosting herbs are expected to enhance the immune system. In this study, we specifically focused on the inflammatory responses of macrophages and T cells isolated from mice that were orally given AME.

Thioglycollate-induced sterile peritonitis was first introduced in 1964 by Gallily et al. and since then has been the most commonly used method for the isolation of primary macrophages [19]. On day 4 after intraperitoneal injection of thioglycollate, the total number of peritoneal exudate cells increases approximately 5-fold [15]. Among these cells, macrophages are the predominant cell type, followed by eosinophils [15]. The source of the increased number of peritoneal macrophages in thioglycollate-injected mice is bone marrow-derived blood monocytes [15]. Upregulation of scavenger receptors occurs during the process of monocyte-to-macrophage differentiation [16–18]. Scavenger receptors, one type of macrophage innate receptors, are responsible for phagocytosis and specifically recognize polyanionic ligands [20]. We used CD11b and several scavenger receptor markers to identify monocyte-derived macrophages in peritoneal exudate cells and found that the CD11b(+)SRA(+) cell population was significantly increased in the AME group. This suggests that administration of AME promotes recruitment and differentiation of blood monocytes to macrophages in response to thioglycollate.

LPS is recognized by the toll-like receptor (TLR)-4/MD-2 complex. TLR4 induces inflammatory responses through two adaptor molecules, MyD88 and TRIF [21]. The MyD88-dependent signaling pathway activates NF-*κ*B and mitogen-activated protein kinase (MAPK) to induce inflammatory genes such as TNF-*α* and IL-6 [22]. The TRIF-dependent signaling pathway activates interferon regulatory factor-3 to produce IFN-*β*, which is required for the upregulation of costimulatory molecules [23, 24]. The TRIF signaling pathway also participates in the activation of NF-*κ*B and MAPK but in a delayed manner relative to the MyD88-dependent pathway [22]. Upregulation of costimulatory molecules is solely TRIF-dependent while inflammatory responses are co-dependent on MyD88 and TRIF [24]. There was no inhibitory effect on the inflammatory markers tested in macrophages from the AME group. Instead, CD86 expression was decreased. CD86 on macrophages binds CD28 on Th cells to strengthen the activity of Th cells [1]. Our results indicate that oral administration of AME does not affect NF-*κ*B- and MAPK-dependent inflammatory responses in macrophages but specifically interferes with the TRIF-dependent pathway that leads to CD86 expression only. Further studies are needed to evaluate whether AME causes alterations in a pathologic situation where macrophages and Th cells predominate.

The LPS-stimulated macrophage system is a very common* in vitro* model for evaluating the anti-inflammatory activity of natural products or drug candidates. Using this model, it is easy to obtain the desired result with lipid-soluble components because they can easily penetrate the cell membrane. Our data showed that peritoneal macrophages isolated from mice that were orally given AME did not show anti-inflammatory effects* ex vivo*, contradicting previously reported* in vitro* results [9, 10]. In contrast, anti-inflammatory activity of AME was observed in the serum response of TNF-*α* and IL-6 upon intraperitoneal injection of LPS. This systemic anti-inflammatory activity is least likely to be mediated by the modulation of macrophages. One of the differences between the* in vivo* and* in vitro* conditions is that LPS is carried in the circulation by several lipoproteins and then cleared by hepatocytes* in vivo*, whereas this event cannot be mimicked* in vitro* [25, 26]. LPS clearance can prevent overstimulation of the liver macrophages [26]. Whether the systemic anti-inflammatory activity of AME is related to LPS clearance in the liver remains to be determined. A similar result was obtained in peritoneal macrophages isolated from mice given oral* Astragalus membranaceus* water extract (unpublished data).* Astragalus membranaceus* and AM belong to the same Qi-tonifying herb category. At this time, we do not know whether* in vivo *anti-inflammatory activity that does not involve macrophage modulation is unique to these medicinal plants or a common property inducible by Qi-tonifying medicinal plants, and we need to accumulate more data to draw any conclusions. In addition, Li et al. reported that atractylenolide I and 14-acetoxy-12-senecioyloxytetradeca-2*E*,8*E*,10*E*-trien-4,6-diyn-1-ol, a type of polyacetylenic compound isolated from AM, have a molecular structure that interacts with membrane-bound glucocorticoid receptor [11]. According to their study, 300 mg/kg of oral atractylenolide I and 30 mg/kg of oral polyacetylene were the minimum doses required to show anti-inflammatory effects [11]. The amount of atractylenolide I in 2,500 mg/kg AME is merely 0.097 mg/kg, a dose far below the minimum required. Moreover, loss of polyacetylenes must have occurred during AME preparation. It is possible that AME contains unidentified glucocorticoid-like compounds that contribute to its systemic anti-inflammatory activity.

A sufficient number of T cells is required to maintain a proper immune response. Under normal conditions, the total T cell number is maintained by the generation of naïve T cells in the thymus and the turnover of peripheral naïve T cells and memory T cells. Mice and humans undergo thymus atrophy with age and accordingly naïve T cell output declines in both species [27, 28]. However, in terms of naïve T cell maintenance, mice produce naïve T cells during their lifetime, whereas adult humans maintain this population by peripheral naïve T cell division [29]. Besides, the lifespan of mouse naïve T cells is 40-fold shorter than their human counterparts [30]. Memory T cells are maintained by intermittent division [31]. The precise survival and homeostatic proliferation mechanism of naïve and memory T cells is not completely defined but involves signals from TCR/MHC complex and cytokines such as IL-7 and IL-15 [31, 32]. The prolonged effect of vaccines depends on memory T cells whereas treatment of lymphopenic conditions requires naïve T cells. We did not determine whether the splenic CD4 T cell population that increased upon AME treatment consisted of naïve CD4 T cells or memory CD4 T cells. A detailed characterization of the cell fraction that responds to AME will help to specify which situation is better suited for the application of AME.

Of note, concurrent upregulation of MHC class II molecules in the spleen occurred in the AME group. MHC class II molecules are necessary to provide antigens to CD4 T cells. We routinely found that the majority of MHC class II expressing cells in the spleen are B cells and the remaining cells are macrophages and dendritic cells. We did not clarify which types of cells showed upregulation of MHC class II molecules after AME administration. Nonetheless, increases in both CD4 T cell number and MHC class II molecule expression in the spleen indicate that supplementation of AME contributes to the systemic maintenance of CD4 T cells. The role of IL-4 under physiological conditions is to enhance the antibody response by promoting the survival and proliferation of B cells and provide defense against helminth infection [33–35]. Splenocytes from the AME groups showed increased IL-4 production during T cell activation* ex vivo* concurrent with decreased IFN-*γ* production. These results suggest that under normal conditions AME promotes the Th2 response. In contrast, oral administration of AM-derived glycoprotein promotes the Th1 response while decreasing the Th2 response in an allergic model [36]. It is not clear whether this compound represents the entire activity of AM. Further study is required to determine whether AME prevents or aggravates pathologic Th2 responses.

## 5. Conclusion

In this study, we observed changes in the responses of macrophages and T cells in normal mice following oral administration of AME. AME enhanced thioglycollate-induced monocyte differentiation in the peritoneum and suppressed LPS-induced TNF-*α* and IL-6 levels in serum. Unlike these systemic anti-inflammatory effects, anti-inflammatory effects were not evident in macrophages isolated from the AME group except for alterations in the expression of costimulatory molecules. AME also influenced the adaptive immune system by increasing the number of CD4 T cells and the expression of MHC class II molecules and promoting the Th2 response over the Th1 response.

## Data Availability

The data used to support the findings of this study are available from the corresponding author upon request.

## Abbreviations

AM: Atractylodes macrocephala Koidz
AME: AM water extract
LPS: Lipopolysaccharide
TNF-*α*: Tumor necrosis factor-*α*
IL: Interleukin
APC: Antigen presenting cell
TCR: T cell receptor
MHC: Major histocompatibility complex
Th cell: T helper cell
DW: Deionized water
FBS: Fetal bovine serum
PBS: Phosphate buffered saline
iNOS: Inducible nitric oxide synthase
COX-2: Cyclooxygenase-2
TLR: Toll-like receptor
IFN-*γ*: Interferon-*γ*
MAPK: Mitogen-activated protein kinase.
